# Efficacy of xenogeneic collagen matrix in the treatment of gingival recessions: a controlled clinical trial

**DOI:** 10.1590/1807-3107bor-2024.vol38.0111

**Published:** 2024-11-08

**Authors:** Karyna de Melo Menezes, Samuel Batista Borges, Isadora Medeiros, Gabriela Ellen da Silva Gomes, Angelo Giuseppe Roncalli, Bruno César de Vasconcelos Gurgel

**Affiliations:** (a) Universidade Federal do Rio Grande do Norte – UFRN, Department of Dentistry, Natal, RN, Brazil.

**Keywords:** Biocompatible Materials, Gingival Recession, Clinical Trial, Surgical Flaps

## Abstract

This study aimed to evaluate the efficacy of a xenogeneic collagen matrix (XCM) in treating gingival recessions (GR) in a thin gingival phenotype. This double-blind, planned, controlled, split-mouth clinical trial included 30 patients with bilateral recessions, randomly assigned to a test group (extended flap + XCM) and a control group (extended flap + connective tissue graft; CTG). Root coverage at 18 months was 1.75 ± 0.8 mm (72.9%) and 2.4 ± 0.51 mm (88.9%) in the test and the control groups, respectively. The upper limit of the confidence interval was not greater than the non-inferiority margin of 0.69 mm. The increase in gingival thickness was greater for autogenous graft (p = 0.003). Both treatments improved quality of life at 18 months. The keratinized tissue width (KTW) increased significantly in the grafted teeth, in both the test (p < 0.001) and the control groups (p < 0.001). Total root coverage was similar in both groups, reaching 70% and 66.7% in the control and test groups, respectively, with no significant differences observed for partial or complete root coverage (CRC). An association was observed in the quality of the gingival phenotype at 18 months according to the treatment group, i.e., a higher percentage of cases with a thicker phenotype was observed in the control group (86.7%), compared with the test group (53.3%) (p = 0.005). XCM was effective in treating GR, but CTG had better results because of significantly increased gingival thickness and phenotypic conversion.

## Introduction

Gingival recession (GR) is the apical displacement of the gingival margin, leading to root surface exposure. It can be caused by different conditions or pathologies, and is associated with loss of clinical attachment.^1^ GR is often associated with aesthetic complaints, root hypersensitivity, difficulty in achieving ideal control of biofilm accumulation, and greater susceptibility to root caries.^2^ These aspects can have an impact on aesthetics, function, and comfort.^3^ Predisposing factors can contribute to the appearance and progression of GR, such as thin periodontal phenotype and lack of keratinized tissue.^4^ A thin phenotype increases the risk of GR, and is more likely to cause greater recession injuries.^5^ Therefore, treatments are required to cover the exposed root and increase the soft tissue volume, since thicker soft tissue and marginal gingiva stability can minimize the risk of GR recurrence^6^.

A connective tissue graft (CTG), combined with a coronally advanced flap, is considered the gold standard for GR treatment.^7^ This approach can offer greater long-term predictability of the root coverage rate, and is widely used for treating a thin phenotype.^8^ Although this option is the first choice for improving a clinical outcome, not all patients accept it,^9^ for the following reasons: a second surgical site is needed,^10^ a limited amount of tissue may be available, and there is increased morbidity, prolonged surgical time, and possible post-surgical complications, such as pain, bleeding, numbness and changes in sensitivity in the donor area.^11^ Accordingly, alternative soft tissue graft materials have been introduced in the field, and have shown favorable results.^12^

Xenogenic collagen matrix (XCM) is a three-dimensional membrane composed of two functional collagen layers (one dense and the other spongy) that provide space for clot formation and growth of adjacent tissue.^12^ XCM has been shown to create sufficient keratinized tissue width (KTW)^9^ and to cover single^13,14^ and multiple GR^15-17^ in controlled and randomized clinical trials. More recently, XCM therapy has been likened to free gingival graft therapy, even after 6+ years of follow-up.^17^ Trial results have shown that XCM promoted long-term improvements in GR reduction and clinical attachment level (CAL).^17,18^ Therefore, this randomized clinical trial (RCT) aimed to evaluate the clinically effective non-inferior XCM for root coverage of GR, compared with CTG, for thin gingival phenotypes, with 18 months of follow-up.

## Methods

### Study design

The present study was a split-mouth, double-blind, longitudinal and prospective RCT. It was approved by the Institutional Ethics Board of the Federal University of Rio Grande do Norte (1.719.095/2016/CAAE-54048516.9.0000.5292) (NCT02980055), and conducted in compliance with the Helsinki Declaration of 1975, as revised in 2013. All patients were informed of the risks and benefits of participation, and all participants signed a written informed consent form.

### Study population and sample size calculation

Fifty-nine eligible patients from the Department of Dentistry, Federal University of Rio Grande do Norte (Natal, RN, Brazil), were randomized and received treatment between February 2017 and October 2019. This study followed CONSORT (Consolidated Standards of Reporting Trials) guidelines.^19^ Inclusion criteria were bilateral RT1-type^20^ on vital upper canines or premolars; without restorations on the root surface; with a thin gingival phenotype, characterized by the transparency of a periodontal North Carolina probe (PCPUNC 15^®^ Hu Friedy, Chicago, USA) introduced into the gingival sulcus;^21^ good periodontal health, characterized by bleeding on probing (BOP) < 10%^22,23^ and probing depth (PD) ≤ 3 mm;^24^ adult > 18 years old; good systemic health. Exclusion criteria were untreated periodontitis; smoking; pregnancy; ongoing orthodontic treatment; contraindication for periodontal surgery; medications known to interfere with periodontal healing; clinically significant dental malposition; and positive history of periodontal surgery in the region.

The sample size calculation (60 sample units required) was based on an estimated difference of 0.69 mm between the test and the control groups for residual GR after root coverage, applying a power (1-beta) of 80% and a one-sided 95% confidence interval.

### Clinical and radiographic assessments

Absence of interproximal bone loss was confirmed with periapical radiographs. Only one tooth on each side with RT1-type^20^ was chosen for root coverage. Graft-type randomization was performed by a computer-generated random sequence shortly before the start of surgery.

PD, GR, CAL and BOP were measured for teeth diagnosed with RT1-type,^20^ as well as the teeth distal and mesial to the tooth submitted to root coverage. In addition, KTW and gingival thickness (GT) were measured for the tooth selected to be grafted. A blinded examiner (S. B. B.), calibrated for PD, GR, KTW and GT measurements by the intraclass correlation coefficient (ICC = 0.655; 0.722; 0.634 and 0.936, respectively), evaluated all the parameters using the University of North Carolina periodontal probe. GT was evaluated by transgingival probing using an endodontic spacer and a silicone stop (Dentsply Maillefer, São Paulo, Brazil), positioned and pressed at the buccal point of the keratinized tissue, between 1.5 to 2 mm from the gingival margin to the resistance of the bone tissue or the tooth.^25^ Subsequently, this measurement was transferred to a digital caliper (Super Caliper, Mitutoyo, Japan), and the data were recorded.

The periodontal health condition was analyzed by collecting data at baseline, 6, 12 and 18 months after therapy, at six sites per tooth (mesiobuccal, buccal, distal-buccal, mesio-lingual/palatal, lingual/palatal and distal-lingual/palatal). For statistical purposes, the average of the three buccal sites was calculated to represent the value of each variable (mesiobuccal, buccal and distal-buccal sites).

### Patient-centered outcomes

The Oral Health-Related Quality of Life (OHRQoL) instrument was applied to assess quality of life^26^. This instrument, with 16 items and responses to each item, indicates the impact of oral health on an individual, ranging from "very bad" (score 1) to "very good" (score 5). The responses were then summed up to give the total score, and the assessed quality of life corresponded to the physical, social and psychological dimensions. A lower score indicates poorer OHRQoL. This assessment was carried out for all the individuals at baseline, 6, 12 and 18 months of follow-up.

### Pre- and intra-surgical procedures

After inclusion, all the patients were evaluated for their oral hygiene condition. When necessary, they were submitted to scaling and root planing sessions, using curettes (Gracey curettes, Hu Friedy, Chicago, USA), two weeks before the surgical procedure. Prophylaxis and oral hygiene advice were also given, with instructions not to use excessive force during brushing, especially of the teeth with GR.

Immediately before surgery, the left and right sides were randomized by a simple random draw by a computer-generated random sequence to receive the CTG or the XCM (Mucograft^®^, Geistlich Pharma AG, Wolhusen, Switzerland). The CTG was harvested from the palate on the same side as its receptor site by using the single linear incision technique.

The coronally advanced flap (CAF) surgical technique^27^ was adopted in both groups, by performing two vertical releasing incisions, as described by Langer and Langer,^28^ but with an extended full-thickness flap up to the mucogingival junction, and partial thickness thereafter, for the distal and mesial teeth immediately adjacent to the tooth diagnosed with the GR to be grafted. All the surgeries were performed by an experienced surgeon (K. M. M.) with a 15c scalpel blade (Swann-Morton^©^, Sheffield, UK), and the portion of the root exposed to the oral environment was carefully planed with a metal curette (Gracey 5-6 Mini-Five) and/or a finishing composite resin drill (Microdont^®^, São Paulo, Brazil), to reduce the root surface convexity.

The harvested CTG was adapted on the root surface in the control group (CAF + CTG), and on the opposite side in the XCM test group (CAF + XCM). Autogenous and xenogenic grafts were fixed with 5.0 nylon simple sutures (Ethicon^®^ - Johnson & Johnson, São Paulo, Brazil) in both groups. The suspending sutures of the coronally positioned extended flaps were performed with the same thread on the teeth with the grafts, and on the distal and mesial teeth. Therefore, the surgical technique and the suture technique were exactly the same for both groups. A synthetic 4.0 silk suture thread (Ethicon^®^ - Johnson & Johnson, São Paulo, Brazil) was used for the donor site. Palate sutures and receptor sites were removed after 07 and 15 days, respectively.

The patients were instructed not to brush the grafted area for two weeks after surgery, and to rinse with 0.12% chlorhexidine gluconate (Colgate, Colgate-Palmolive Company, São Bernardo do Campo, Brazil) twice a day for 15 days. All the volunteers took 1 tablet of 4 mg dexamethasone, and 1 capsule of 500 mg amoxicillin one hour before the surgical procedure. After surgery, they took one 500 mg amoxicillin capsule every 8 hours for a day, one 100 mg nimesulide tablet every 12 hours for 3 days, and one 500 mg dipyrone tablet every 6 hours for 3 days.^29^

### Statistical analysis

The data were analyzed using descriptive and inferential statistics, with non-parametric and parametric tests, using the Statistical Package for Social Sciences (SPSS software, v. 23.0, free version, SPSS, Chicago, USA). The t-test for independent samples was performed to analyze the statistical difference among the surgery-related independent variables. The Friedman, Wilcoxon and Mann-Whitney tests were performed to determine the statistical differences for the main clinical parameters (PD, GR, CAL, KTW and BOP), and to identify at what follow-up time this difference occurred. Analysis of variance (one-way ANOVA) was performed for GT.

The chi-square test (x^2^) was used to assess the possible association between root coverage (RC) rate and gingival phenotype (GP), according to the independent variables. The McNemar test analyzed the changes in GP, while the ANOVA test for repeated measures with the Bonferroni post-test observed the change in the quality of life of individuals. The significance level was set at 5%.

## Results

**Figure 1:**
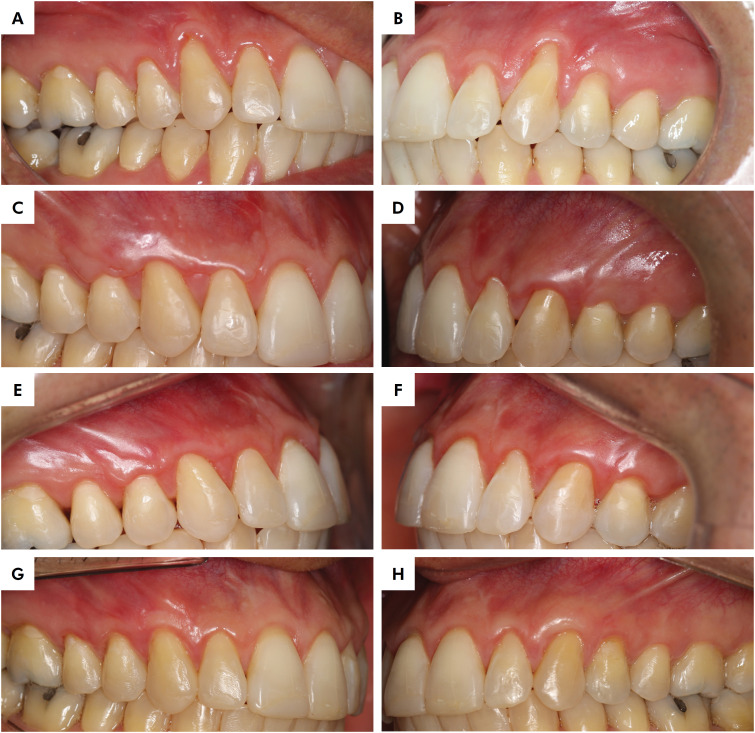
Representative clinical case of both treatment groups. Baseline views of test (A) and control (B) groups. Six-month follow-up of XCM (C) and CTG (D). Twelve-month follow-up of XCM (E) and CTG (F). Eighteen-month postoperative outcome of XCM (G) and CTG (H).

Fifty-nine individuals sought treatment for GR, but only 51 were identified with recessions. Four individuals refused to participate after the treatment protocol was explained. Fifteen individuals were excluded for not meeting the inclusion criteria. Thirty-two patients started the study according to its split-mouth design, and their bilateral GR was randomized into two groups: test group (CAF + XCM) and control group (CAF + CTG). Two individuals were lost during the follow-up. Thus, 30 patients, 15 male and 15 female, mean age of 30.3 years (± 6), were evaluated at a follow-up of up to 18 months (Figure 1).

**Figure 2:**
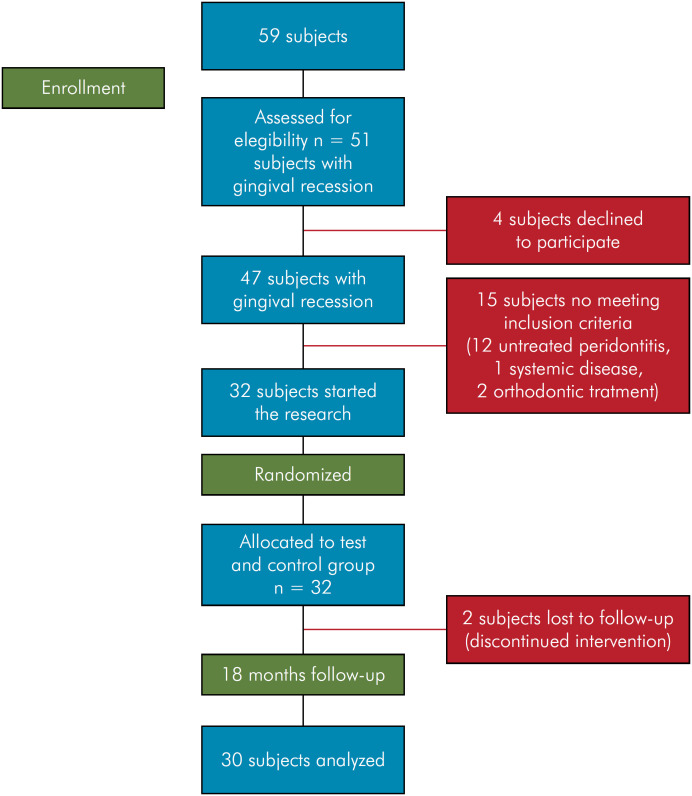
CONSORT flow diagram of the study.

All the grafts were placed in the maxilla. The highest prevalence of GR was found in the first premolars (30; 50%), followed by the canines (25; 41.7%) and the second premolars (5; 8.3%). A total of 60 teeth with GR were treated with CTG or XCM. Post-surgical complications were observed in 2 patients, owing to hemorrhage at the donor site. Table 1 shows the surgery time (in minutes) for both groups (p < 0.001), the mesiodistal width of the GR, the length and height of the grafts (p > 0.05), and the thickness of the XCM (p < 0.001).

**Table 1:** Description of the quantitative data. *t* test for independent samples.

Variable	Test group			Control group			p-value
	Mean ± SD		Min– Max	Mean ± SD		Min– Max	
Surgery time (min)	45.6	6.9	56–106	75.5	11.4	29–55	< 0.001
Mesiodistal width of the GR (mm)	3.4	0.72	2–5.7	3.6	0.83	1.9–4.5	0.232
Graft thickness (mm)	3.37	1.07	0.62–1.53	1.13	0.23	1.2–5.8	< 0.001
Graft length (mm)	10.5	1.87	8–14	10.4	1.59	5–14	0.268
Graft height (mm)	7.0	1.27	4–14	6.6	2.16	5–9	0.119

GR: gingival recession; SD: Standard deviation; min: minutes; mm: millimeters.

There were no statistical differences at any follow-up period for PD or POB, either for the grafted tooth or the mesial and distal teeth involved in the extended flap. KTW was significantly augmented in the grafted teeth, in both the test (p <0.001) and the control (p < 0.001) groups, and presented increases from baseline to 6, 12 and 18 months (Table 2). The grafted teeth in both the test and the control groups showed statistically significant reductions in GR (p < 0.001), and a gain in the CAL (p < 0.001), comparing the baseline periods of 3 and 6 months, with no significant difference between the treatment groups (Table 2).

**Table 2:** Comparison of clinical parameters. Friedman, Wilcoxon, Mann-Whitney tests and ANOVA.

Variable	Test group					Control group					p2
	Mean ± SD		Md	Min–Max	Q_25_–Q_75_	Mean ± SD		Md	Min–Max	Q_25_–Q_75_	
PD mm t0	2	0.41	1.9	1.3–2.8	1.7–2.3	2	0.35	2	1.7–2.7	1.7–2.3	0.612
PD mm t6	1.8	0.37	1.7	1–2.7	1.7–2	2	0.41	1.8	1.3–2.8	1.7–2.2	0.124
PD mm t12	1.7	0.38	1.5	1–2.4	1.6–1.9	1.9	0.43	1.8	1.2–2.8	1.7–2.3	0.132
PD mm t18	1.8	0.42	1.6	0.9–2.3	1.4–2	1.9	0.41	1.9	1.2–2.7	1.8–2.2	0.211
p1	0.063					0.612					
GR mm t0	2.4ª	0.98	2	1–5	1.9–3	2.7^a^	1.24	2	1–5	2–4	0.460
GR mm t6	0.6^b^	0.82	0	0–3	0–1	0.3^b^	0.55	0	0–2	0–0.6	0.211
GR mm t12	0.62^b^	0.79	0	0–3	0–1	0.3^b^	0.53	0	0–2	0–0.5	0.223
GR mm t18	0.65^b^	0.80	0	0–3	0–1	0.3^b^	0.51	0	0–2	0–0.5	0.218
p1	<0.001					<0.001					
CAL mm t0	3.9^a^	1.29	3.9	2–7	3–4.6	4.2ª	1.45	4	2–7.3	3–5	0.379
CAL mm t6	1.9^b^	0.86	2	1–4	1–2.8	2^b^	1.10	1.9	1–5	1–3	0.848
CAL mm t12	1.9^b^	0.86	2	1–4	1–2.8	2^b^	1.10	1.9	1–5	1–3	0.845
CAL mm t18	1.9^b^	0.86	2	1–4	1–2.8	2^b^	1.10	1.9	1–5	1–3	0.812
p1	<0.001					<0.001					
KTW mm t0	3.3^a^	1.3	3	1.5–7	2–3.6	3.3^a^	1.42	3	2–7.5	2–4.5	0.892
KTW mm t6	4^b^	1.3	4	2–7	3–5	4.6^b^	1.3	4	3–7	4–5.3	0.073
KTW mm t12	4^b^	1.34	4	2–6.9	3–4.9	4.5^b^	1.42	4	3–7	4–5.2	0.068
KTW mm t18	4.2^b^	1.32	4	2–6.8	3–5	4.6^b^	1.40	4	3–7	4–4.9	0.071
p1	<0.001					<0.001					
BOP t0	0.67	0.88	0	0–3	0–1	0.60	0.93	0	0–3	0–1	0.581
BOP t6	0.47	0.82	0	0–3	0–1	0.43	0.86	0	0–3	0–0.25	0.675
BOP t12	0.38	0.78	0	0–2	0–1	0.39	0.87	0	0–3	0–0.22	0.645
BOP t18	0.35	0.74	0	0–2	0–2	0.41	0.74	0	0–2	0–0.19	0.545
p1	0.466					0.425					
	Mean ± SD		SE	Min-Max	CI (95%)	Mean ± SD		SE	Min-Max	CI (95%)	p2
*GT mm t0	0.83^a^	0.21	0.38	0.44–1.28	0.75–0.91	0.89^a^	0.06	0.57	0.50–1.92	0.77–1	0.401
*GT mm t6	1.07^b^	0.31	0.57	0.56–1.92	0.96–1.19	1.37^a^	0.39	0.76	0.68–2.53	1.21–1.52	0.003
*GT mm t12	1.08^b^	0.32	0.51	0.56–1.92	0.96–1.19	1.42^a^	0.4	0.74	0.68–2.5	1.21–1.52	<0.001
*GT mm t18	1.1^b^	0.4	0.55	0.56–1.92	0.96–1.19	1.45^a^	0.41	0.72	0.68–2.51	1.21–1.52	<0.001
p1	<0.001					<0.001					

t0: baseline; t6: 6 months; t12: 12 months; t18: 18 months; PD: Probing depth; GR: Gingival recession; CAL: Clinical attachment level; KTW: Keratinized tissue width; BOP: Bleeding on probing; GT: Gingival thickness. Different lowercase letters indicate a significant difference.

* Variable with normal distribution.

The difference in the average GR in the grafted teeth, between baseline and 6 months was used to calculate the final RC percentage in both treatment groups. The averages of 2.4 and 2.7 mm of GR at baseline (start of the study) became 0.65 mm and 0.3 mm of residual recession after 18 months for the test and the control groups, respectively. Thus, the average RC percentages were 72.9% and 88.9% for the test and the control groups, respectively, without any statistical difference (p > 0.05). Complete root coverage (CRC) was similar for both groups, and achieved in 70% and 66.7% of the cases in the control and the test groups, respectively, with no significant differences for partial RC or CRC (p = 0.781). All the teeth with partial coverage bone dehiscence were detected after the flap elevation (Table 3).

**Table 3:** Root coverage and gingival phenotype at 18 months, according to independent variables of interest percentage (x^2^ test).

Variable		Root coverage			Gingival phenotype at t18		
		Partial (%)	Total (%)	p-value	Thin (%)	Thick (%)	p-value
Treatment group							
	Test	10 (33.3)	20 (66.7)	0.781	14 (46.7)	16 (53.3)	0.005
	Control	9 (30)	21 (70)		4 (13.3)	26 (86.7)	
Sex							
	Female	13 (43.3)	17 (56.7)	0.052	10 (33.3)	20 (66.7)	0.573
	Male	6 (20)	24 (80)		8 (26.7)	22 (73.3)	
Age (years)							
	18–30	14 (38.9)	22 (61.1)	0.141	13 (36.1)	23 (63.9)	0.206
	> 30	5 (20.8)	19 (79.2)		5 (20.8)	19 (79.2)	
Keratinized tissue width of t18							
	> 3 mm	6 (37.5)	10 (62.5)	0.558	6 (37.5)	10 (62.5)	0.445
	< 3 mm	13 (29.5)	31 (70.5)		12 (27.3)	32 (72.7)	
Previous orthodontic appliances							
	Yes	16 (50)	16 (50)	0.001	12 (37.5)	20 (62.5)	0.175
	No	3 (10.7)	25 (89.3)		6 (21.4)	22 (78.6)	
Time using an orthodontic appliance (months)							
	> 42	9 (45)	11 (55)	0.465	6 (30)	14 (70)	0.258
	< 42 months	7 (58.3)	5 (41.7)		6 (50)	6 (50)	
Toothbrush type							
	Extra soft or soft	10 (25)	30 (75)	0.116	12 (30)	28 (70)	1.000
	Medium or hard	9 (45)	11 (55)		6 (30)	14 (70)	

T18: 18 months.

In addition, the CRC rate was higher when the patient did not use an orthodontic appliance previously (89.3%) (p = 0.001) (Table 3). An association was observed in the quality of the GP at 18 months according to the treatment group, i.e., a higher percentage of cases with a thicker phenotype was observed in the control group (86.7%), compared with the test group (53.3%) (p = 0.005) (Table 3). Moreover, the change in phenotype was assessed, and revealed that the grafted teeth (16 teeth in the test group [53.3%] and 26 in the control group [86.7%]) showed a thick phenotype at 18 months (Table 4).

**Table 4:** Change in the gingival phenotype (GP). McNemar test.

Variable		Change in the GP				
		GP - t0 to GP t6		p-value 1	GP - t0 to GP t18	p-value 2
Test group						
	Thin	30 (100%)	14 (46.7%)		14 (46.7%)	
	Thick	0 (0%)	16 (53.3%)	< 0.001	16 (53.3%)	< 0.001
Control group						
	Thin	30 (100%)	4 (13.3%)		4 (13.3%)	
	Thick	0 (0%)	26 (86.7%)	< 0.001	26 (86.7%)	< 0.001

p1 and p2 values indicate a *significant difference* of change in the GP from baseline to 6 months, and baseline to 18 months, respectively. t0: baseline; t6: 6 months; t18: 18 months.

Assessing the quality of life using the OHRQoL instrument showed a significant improvement in the patients’ quality of life, and an increase in the sum of the means and medians of the physical, social, and psychological domains, referring to improvements in quality of life. Changes in quality of life were observed when comparing the baseline with 6, 12 and 18 months (p < 0.001) (Table 5).

**Table 5:** Quality of life analysis and its domains. ANOVA test for repeated measures

Variable		Mean ± SD		Md	Min-Max	Q_25_-Q_75_	p-value
Physical domains							
	t0	21.3^a^	0.88	20.5	12–29	18–25.25	
	t6	26.8^b^	0.56	28.0	20–30	20.5–28.50	< 0.001
	t12	27.6^b^	0.51	29.0	22–30	24.7–29.5	
	t18	26.9^b^	0.51	27.5	22–30	24.5–30.0	
Social domains							
	t0	18.5^a^	0.82	19.0	9–25	15.25–20.25	
	t6	21.9^b^	0.50	22.0	16–25	19.0–23.0	< 0.001
	t12	22.4^b^	0.37	22.5	20–25	21.0–25.0	
	t18	22.6^b^	0.41	23.0	20–25	21.0–25.0	
Psychological domains							
	t0	18.1^a^	0.84	19.0	11–25	15.25–21.0	
	t6	22.0^b^	0.50	22.0	17–25	20.0–25.0	< 0.001
	t12	22.2^b^	0.42	23.5	18–25	20.5–25.0	
	t18	21.7^b^	0.47	23.0	17–24	21.5–25.0	

Different lower-case letters indicate a significant difference. t0: baseline; t6: 6 months; t12: 12 months; 18: 18 months.

## Discussion

The results of this clinical trial provide evidence to support the hypothesis of non-inferiority of the XCM compared with CTG, in terms of better RC, shorter surgical time, and improved quality of life of individuals, as evaluated after 18 months of follow-up. Previous studies have shown that the XCM can be considered an alternative to CTG for GR treatment, but with statistically lower RC rates.^15,30,31^

A recent multicenter clinical trial evaluated multiple recessions, and tested the hypothesis of non-inferiority of the XCM in relation to CTG, using CAF associated with the matrix (test group), and CAF associated with connective tissue (control group). The results of the study by Tonetti et al.^15^ did not support the non-inferiority hypothesis for the XCM, since there was full RC in only 48% (117 teeth) of the test group, versus 70% (170 teeth) of the control group.^15^

In contrast to the results of the above-mentioned study,^15^ the present study showed that XCM was not inferior to CTG, since 66.7% and 70% of total RC was observed in the test and control groups, respectively. The reduction in recession was 1.75 mm in the test group and 2.4 mm in the control group, thereby yielding a difference of 0.65 mm between the treatment groups. If the difference were greater than 0.69 mm, the hypothesis of the non-inferiority of XCM would have been rejected. Furthermore, Tonetti et al.^15^ did not mention the GP quality of the teeth. The current study had a split-mouth design, and used only thin GPs in RT1-type GR. It had the further advantage of allowing a reduction in the variability of the estimated treatment effect among the individuals, hence potentially reducing the required sample size, compared with the parallel group study by Tonetti et al.,^15^ with the same power.^32^

Regarding the biomaterials used, their limitations must be considered. CTG contains living cells, blood vessels, and other constituents, such as collagen. XCM is composed exclusively of collagen; therefore, the repair and incorporation processes of new cells are different from those of CTG. Consequently, the release of incisions far from the GR area could increase the size of the vascular support area, thus preventing exposure of the biomaterial, providing better vascularization,^27^ improving graft nutrition, and favoring RC.^33,34^ This is why the extended flap technique was used in the current study.

The differences in the composition of autogenous and xenogenic grafts may have been responsible for the significant intragroup increase in GT from baseline to 6, 12 and 18 months in both groups (p < 0.001), and for the significant increase found in the control group for the intergroup analysis at 6, 12 and 18 months (p = 0.003 e p < 0.001) (Table 2). In addition, gains of 0.27 mm and 0.56 mm in thickness were observed in the test and the control groups, respectively (Table 2), thus corroborating another study that evaluated GT^16^ after mandibular GR treatment with XCM and CTG. The authors showed that the increase in GT for the control group (CTG; 1.1 mm) was statistically different from that of the test group (XCM; 0.27 mm).

In a recent review, Kim, Bassir and Nguyen^35^ showed that upper anterior teeth presented positive relationships between GP, GT and KTW,^36^ thus revealing that individuals with shorter KTW and thin GP were more likely to have GR, compared with those with greater KTW and thicker GP. Thus, treatment of GR associated with grafts, especially the autogenous graft, can help prevent future GR by increasing the thickness and width of keratinized tissue.

In the present study, different graft thicknesses were used, namely 3.37 mm for XCM and 1.13 mm for CTG. However, both the control group (CTG) and the test group (XCM) demonstrated an increase in GT, as depicted in Table 2, and discussed earlier. This rise in GT in our study resulted in a shift in phenotype quality, by transitioning from thin to thick in 16 teeth (53.3%) in the test group, and 26 teeth (86.7%) in the control group, as detailed in Table 4.

The conversion from thin to thick phenotype may improve the long-term stability of the gingival margin, since thick phenotypes are less likely to develop GR or its recurrence.^4^ Additionally, it has been shown that thin GPs in the receptor site can lead to a higher shrinkage rate, and hence graft retraction^33^. However, when the behavior of these two types of grafts was evaluated in the present trial, a thin GP did not appear to be a limiting anatomical feature for RC, since there were no statistically significant differences for residual GR or CAL (Table 2), or for the number of teeth with full RC at 18 months (20 and 21 teeth in the test and the control groups, respectively) (Table 3). These results suggest that other factors may be related to the partial coverage of teeth with thin GP.

Because of the orthodontic movement or buccal projection of the teeth, the alveolar bone over these teeth can undergo resorption or dehiscence as a way to correct their positioning.^37^ This could contribute toward increasing the prevalence of GR,^38^ and may be the reason for the statistical difference observed in the cases where individuals had undergone previous orthodontic treatment. A higher total coverage rate (89.3%) could be expected in individuals who had never been submitted to orthodontic treatment. In cases where orthodontic treatment took place prior to root covering surgery, only 50% of the cases showed full RC (p = 0.001; Table 3).

A further objective of the present study was to control the possible biases of the variables, such as height of the grafts and medial-distal width of the recession (p < 0.05; Table 1), right at the beginning of the study, to show that the clinical conditions were the same at baseline, thereby asserting that the measurement-related biases were under control. This split-mouth trial showed that the tissues presented the same biological behavior in both groups. However, it is difficult to separate the quality of life assessment results between the test and the control groups to identify which group presented the best results for this analysis. Limited data are available regarding the sample size in split-mouth trials, and a rigorous comparison with parallel group studies found in the literature is also unavailable.^32^ Furthermore, the RC achieved in the test and the control groups should be evaluated for a longer period to assess gingival margin stability and cases of change in the GP. The present study was the first to report these results in a split-mouth RCT.

## Conclusion

Within the limits of the present study, it can be concluded that root coverage improved the clinical parameters and the quality of life of both groups. CTG in the thin gingival phenotype was clinically superior to XCM in terms of final gingival thickness, and resulted in minor gingival recession. Nevertheless, XCM represented a viable alternative at up to 18 months of follow-up.
